# Conserved Expression Patterns Predict microRNA Targets

**DOI:** 10.1371/journal.pcbi.1000513

**Published:** 2009-09-25

**Authors:** William Ritchie, Megha Rajasekhar, Stephane Flamant, John E. J. Rasko

**Affiliations:** 1 Gene and Stem Cell Therapy Program, Centenary Institute, Camperdown, Australia; 2 Faculty of Medicine, University of Sydney, Sydney, Australia; 3 Cell and Molecular Therapies, Sydney Cancer Centre, Royal Prince Alfred Hospital, Camperdown, Australia; Duke University, United States of America

## Abstract

microRNAs (miRNAs) are major regulators of gene expression and thereby modulate many biological processes. Computational methods have been instrumental in understanding how miRNAs bind to mRNAs to induce their repression but have proven inaccurate. Here we describe a novel method that combines expression data from human and mouse to discover conserved patterns of expression between orthologous miRNAs and mRNA genes. This method allowed us to predict thousands of putative miRNA targets. Using the luciferase reporter assay, we confirmed 4 out of 6 of our predictions. In addition, this method predicted many miRNAs that act as expression enhancers. We show that many miRNA enhancer effects are mediated through the repression of negative transcriptional regulators and that this effect could be as common as the widely reported repression activity of miRNAs. Our findings suggest that the indirect enhancement of gene expression by miRNAs could be an important component of miRNA regulation that has been widely neglected to date.

## Author Summary

microRNAs are small RNA molecules that regulate gene expression by controlling the output of proteins and other RNAs. The exact mechanism through which a microRNA binds to its target and how this affects the target is still a subject of much debate. In this article, the authors sought to find a reverse approach to discover the impact of microRNAs on gene expression. Instead of searching for specific targets of a given microRNA, they searched for microRNA signatures: changes in the levels of microRNAs across multiple tissues that impacted significantly the levels of messenger gene expression in these same tissues. Because many core biological functions are conserved between human and mouse, the authors compared these microRNA signatures between these two species. They found that identical microRNA signatures between these organisms could effectively predict microRNA targets and could estimate the global impact of individual microRNAs on gene output. They further demonstrated that many microRNAs act as expression enhancers by inhibiting gene repressors.

## Introduction

microRNAs (miRNAs) are short 20–22 nt long endogenous non-coding RNA molecules that reduce gene expression via degradation of messenger RNA (mRNA) [1] and translational inhibition [2]. These micro managers [3] play essential roles in major biological processes such as cell proliferation and differentiation [4] development [5] and disease [6],[7]. miRNAs can tune the expression of multiple genes including complex networks of transcription factors, signaling pathways [8] and other regulatory loops [9]. It is thought that an essential component of miRNA regulation involves the formation of a duplex between the miRNA and the 3′ untranslated region (3′UTR) of a target mRNA. This duplex is characterized in animals by a perfectly paired seed region at the 5′ end of the miRNA and a more loosely paired 3′ extremity [10]. This property of miRNA targeting has provided the foundation for the majority of algorithms dedicated to target prediction [11]–[13] and has been instrumental in discovering miRNA-target pairs.

We set out to establish a new approach for the identification of miRNA targets based on a comparison of expression data of miRNAs with that of mRNAs. Because miRNAs can reduce the expression level of targeted genes, there should be an inverse correlation between the expression level of a given miRNA and the expression level of its cognate target. Previous related attempts using similar methodologies were successful only when combined with the more classical algorithms cited above [14]. The success of this type of approach has been limited due to high levels of noise inherent in large scale expression profiling of both miRNAs [15] and mRNAs. Additionally, a correlation (or inverse correlation) in expression does not necessarily imply a direct functional relationship between two molecules.

We have devised a novel method for inferring functional relations between miRNAs and mRNAs that relies solely on expression data. These relationships were established independently of binding energy calculations or seed region conservation and may therefore be used to support or temper predictions of existing algorithms. We used conservation between species to mitigate the problem of noisy data. Our procedure detected strong correlations (and inverse correlations) between human miRNA and mRNA expression and consolidated this relation with orthologous mouse miRNA and mRNA expression. We defined conserved negative correlation (CNC) as an inverse relation between the expression level of a miRNA and an mRNA in both human and mouse. Conversely, we defined conserved positive correlation (CPC) as a positive relation between a miRNA and an mRNA in these two organisms.

## Results

### Conserved positive and negative correlations between miRNAs and mRNAs

We sought to infer molecular relationships between specific mRNAs and miRNAs. To achieve this, we collected human and mouse miRNA expression data from the miRNA expression atlas [16], human mRNA expression data from 120 “hgu133a” Affymetrix human microarrays and from 75 “430_2.0” Affymetrix mouse microarrays. In total, after selection of transcripts with sufficient proof of orthology, our dataset contained expression measurements of 117 orthologous miRNAs and 6920 orthologous protein coding genes from 35 different tissue samples in human and 28 in mouse (see Materials and Methods and Tables S1, S2 & S3).

We calculated correlation coefficients for all of the 809,640 (117*6920) miRNA/mRNA pairs. Due to the disparate nature of the two expression measurement technologies used (cloning and sequencing from the Atlas to measure miRNA expression versus hybridization efficiency for the microarrays to measure mRNA expression), we used the non-parametric Kendall's rank correlation distance measurement [17]. For each miRNA/mRNA pair we calculated a correlation coefficient for human and another for mouse. Each pair was considered to be a conserved negative correlation (CNC) pair if the correlation coefficient in both human and mouse was below −0.3. Conversely, each pair was considered to be a conserved positive correlation (CPC) pair if the correlation coefficient in both human and mouse was above 0.3.

### Conserved negative correlation between miRNAs and mRNAs efficiently detects miRNA targets

**Figure 1:**
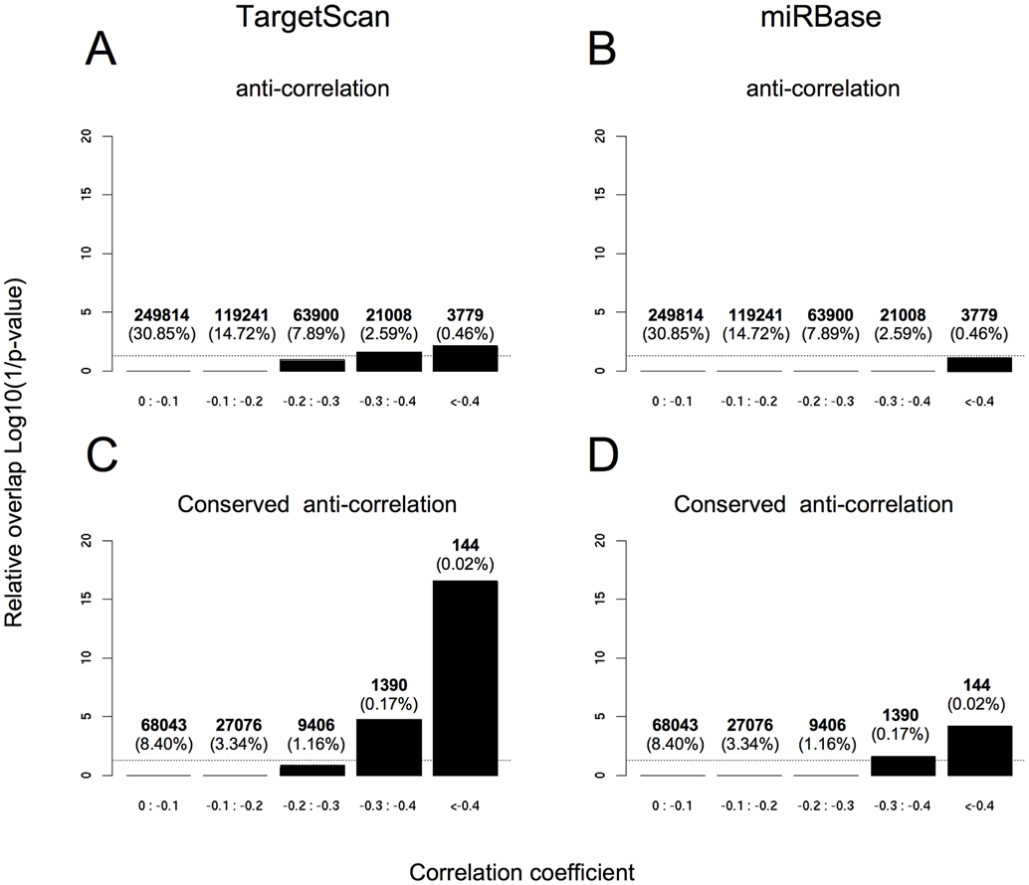
miRNA/mRNA pairs with strong conserved negative correlation coefficients overlap with miRBase and TargetScan predictions. The expression levels of 809,640 miRNA/mRNA pairs were compared across 35 human tissues and a correlation coefficient (*r*) for each pair was calculated. Pairs with a negative *r* (inverse correlation) were binned into 5 groups. The first bin contained pairs for which *r* was between 0 and −0.1 (poor negative correlation) and the last contained pairs for which *r* was below −0.4 (strong negative correlation). Because our analysis identified only one CNC pair with an r below −0.5, no bin was created for this category. For each bin, the relative overlap between these pairs and putative miRNA/target-mRNA pairs predicted by **(A)** TargetScan and **(B)** miRBase was calculated. The degree of overlap between pairs in each bin and pairs from the two databases are represented by vertical bars. The number of pairs in each bin and their percentage of the total number of pairs are shown above these bars. The threshold for significant overlaps (p-value<0.05) is represented by a horizontal dotted line. For example, pairs with negative correlation coefficients between −0.3 and −0.4 show a significant overlap with predictions from TargetScan but not with predictions from miRBase. A similar analysis was conducted in **(C)** and **(D)** except that conserved correlation scores between human and mouse were calculated. Conserved negative correlation scores provided a higher overlap with existing predictions from miRBase and TargetScan.

The binding of a given miRNA to its cognate 3′UTR can lead to degradation of the miRNA. This type of interaction could be detected by miRNA/mRNA pairs that show significant negative correlations in expression. To verify this, we measured the degree of overlap between negatively correlated pairs and predicted target genes from two independent target prediction programs (Figure 1). Each miRNA/mRNA pair was placed in five bins according to their correlation coefficients. Each bin was then compared to miRNA target predictions maintained in two popular databases: miRBase [18] and TargetScan [11]. We performed an enrichment analysis to determine the relative overlap between the predictions made by these two databases and the pairs in each bin (see Materials and Methods). Our hypothesis was that bins with a high level of overlap would be indicative of high confidence miRNA target predictions. This analysis, when conducted solely on human expression data (Figure 1A and 1B) revealed little overlap between negatively correlated pairs and miRNA-target pairs predicted by TargetScan and no significant overlap between negatively correlated pairs and miRNA-targets predicted by miRBase. However, when the same analysis was conducted using expression data from both mouse and human (bottom panels) we observed a significant overlap between conserved negative correlations pairs (CNCs) and predictions from both TargetScan and miRBase even though mRNA genes from CNC pairs did not show higher sequence conservation in their 3′UTR than non conserved pairs (see Text S1). Because our approach relies entirely on expression data and is completely independent from miRBase and TargetScan, the overlap between CNCs and these two databases are likely to represent true functional miRNA-target pairs. These “high-confidence” pairs can be efficiently detected using CNC whereas the use of correlation statistics in only one species fails to achieve significance (Figure S1). Interestingly, the number of CNC pairs overlapped systematically more with TargetScan than miRBase. This could be due to the fact that all TargetScan predictions are based on perfect complementarity of at least 7 or 8 nt between the 3′UTR and the miRNA “seed” region whereas 27% of miRBase predictions show a seed complementarity of 6 or less according to our data. This shorter complementarity could reduce the level of mRNA destabilization [19] and would therefore be more difficult to detect by our method as it is based solely on mRNA levels.

**Figure 2:**
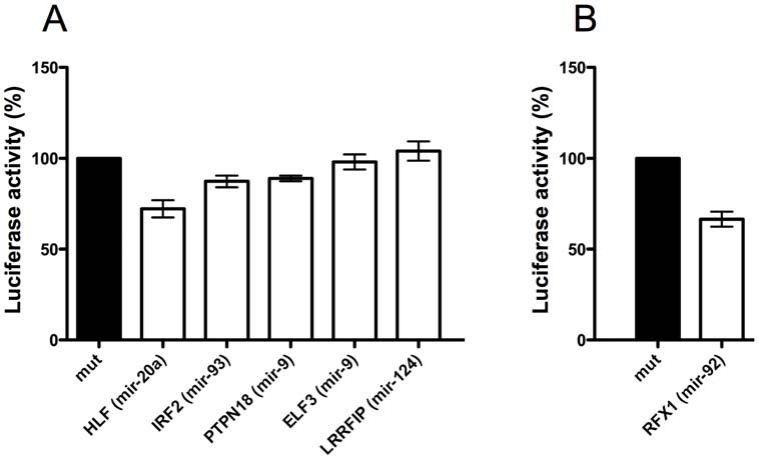
Experimental validation of predicted miRNA targets using a luciferase reporter assay. Histograms showing the luciferase activity of reporter plasmids containing endogenous 3′UTR sequences of indicated genes (white) as a percentage of the activity of the corresponding mutated microRNA seed region (black). The endogenous sequence comprised ∼500 bp of the 3′UTRs inserted in the 3′UTR of the renilla luciferase gene. The mutant sequence was identical to the WT except that it had the predicted miRNA ‘seed’ binding region deleted or mutated. Each of the plasmids was co-transfected with the relevant pri-miRNA.** A)** Of the 5 putative targets discovered by considering high scoring CNC pairs tested, 3 showed significant repression. **B)** The putative target discovered by considering intermediate gene regulation (see text) showed significant repression. Asterisks indicate p-values<0.05, using the non-parametric Mann-Whitney U test.

To further confirm the successful detection of miRNA-target pairs, we randomly selected 5 miRNA/mRNA pairs with correlation coefficients below −0.4 in both species (there were 144 such pairs in our dataset: Table S4). These pairs were tested using a luciferase assay. We compared the level of repression of wild type targets in the native 3′UTR of the gene, to a mutated 3′UTR in which the seed region had been removed or mutated (see Materials and Methods). Surprisingly only 48 human targets confirmed using this luciferase assay have been reported in the miRecords database [20]. Because this assay requires the identification of a miRNA binding site and our method does not detect binding sites but putative functional interactions between miRNAs and mRNAs, we used potential 3′UTR binding sites arising from each CNC pair based solely on a seed region complementarity of 7 nt. If multiple potential sites were identified, we selected the one with the highest binding energy between the miRNA and the 3′UTR of the mRNA. Of the 5 miRNA/mRNA pairs tested, 3 were validated as true miRNA targets (Figure 2A) demonstrating the utility of our approach.

### Using conserved negative correlation to discover new miRNA regulatory mechanisms

One key advantage of our approach is that we are able to discover functional relations between miRNAs and mRNAs without restricting our search to a specific mode of action. Other approaches may be constrained by the strength and location of a miRNA binding to its target. Consequently, we can use these functionally related pairs to test new modes of interaction between miRNAs and mRNAs. For example, we wished to examine whether miRNAs could reduce gene expression by binding to a site other than the 3′UTR. To investigate this possibility we used a technique designated “energy walk” that involves analysis of different regulatory regions of CNC gene pairs to locate regions with high binding potential for miRNAs (see Materials and Methods). Here we used free energy as the sole criterion to identify binding sites because of the importance of miRNA binding energy in target recognition [21],[22]. In our first energy walk, we considered the 1390 CNC pairs with negative correlation scores below −0.3. We examined 5 types of regions: 3′UTR, 5′UTR, coding region, a 3 kb region upstream of the mRNA transcription start site and VISTA [23] predicted enhancer regions flanking the mRNA. For each CNC pair, these 5 regions were scanned once for each miRNA involved in the pair. A hit was recorded if a high-energy binding site (<−20 kCal) between the miRNA and the sequence corresponding to one of the five regulatory regions was found. We then randomly shuffled the miRNAs and mRNAs in each pair and performed an energy walk on these shuffled pairs. For each region, we tested if there was a difference in the number of high-energy binding sites (<−20 kCal) between the CNC pairs and the shuffled pairs using Fisher's exact test. The results of this test (Table 1A) showed that CNC pairs had a significantly higher number of binding sites in the 3′UTR (p-value = 6e-5) compared to the shuffled control. This result demonstrated, as previously predicted by other bioinformatics analysis [24], that a large number of miRNAs that inhibit mRNA expression do so by binding to the 3′UTR. Interestingly, the coding region exhibited a high number of binding sites of borderline significance (p-value = 0.07) suggesting that a minority of miRNAs could possibly bind to elements of the coding region and inhibit mRNA expression as has been recently suggested [25]. The number of binding sites in the 3 other regions did not differ between the CNC pairs and the shuffled pairs. This suggests that miRNAs are unlikely to regulate mRNA expression by binding directly to enhancers, promoters or 5′UTRs.

**Table 1:** Energy walk across 5 potential regulatory regions for CNC and CPC pairs.

**A**	**CNC pairs**		**Enhancers** *	**TSS** *	**5′UTR**	**coding**	**3′UTR**
		N° binding sites (CNC pairs)	2857	1672	535	834	958
		N° binding sites (shuffled pairs)	2925	1579	496	746	743
		**P-value (real vs shuffled)**	**0.45**	**0.21**	**0.32**	**0.07**	**6.00E-005**
**B**	**CPC pairs**		**Enhancers** *	**TSS** *	**5′UTR**	**coding**	**3′UTR**
		N° binding sites (CPC pairs)	2426	1973	738	1013	948
		N° binding sites (shuffled pairs)	2520	2095	690	1081	889
		**P-value (real vs shuffled)**	**0.25**	**0.13**	**0.29**	**0.24**	**0.27**

*: For these regions, both strands were examined, explaining the higher number of binding sites.

For each miRNA/mRNA of a CNC (**A**) pair and CPC pair (**B**), we analyzed 5 predicted regulatory regions of the mRNA for enrichment in binding sites for the corresponding miRNA. The number of sequences from each of these 5 regions containing high energy binding sites (number of high energy binding sites in CNC and CPC pairs) for the miRNA was recorded. The miRNA/mRNA pairs were then shuffled, each miRNA reassigned to a randomly selected mRNA. The same analysis was performed on this control set (number of high energy binding sites in shuffled pairs). By comparing the number of high energy binding sites in the CNC and CPC pairs with the number of high energy binding sites in the same number of shuffled pairs for each region, we were able to find regions that were significantly enriched in binding sites for miRNAs. This comparison was done using Fisher's exact test for categorical data with p-values<0.05 defined as significant.

### Using conserved positive correlation to investigate up-regulation by miRNAs

It has been suggested that miRNAs can increase gene expression by binding to promoter regions [26] or the 5′UTR of viral genes [27]. To examine this phenomenon, we reanalyzed 10 published microarray experiments in which a miRNA had been transfected into cells *in vitro*. We noted that the number of under- and over-expressed mRNAs after transfection was comparable (Table S5) which may be the consequence of endogenous miRNA saturation after transfection [28] or may suggest that miRNAs serve an equally important role in gene repression and induction. To further explore increased mRNA expression consequent to miRNAs, we studied the 1717 non-adjacent CPC pairs with a correlation coefficient above +0.3 (see Materials and Methods). The energy walk was used to identify regions that were preferentially targeted by miRNAs that increase mRNA expression (Table 1B). No CPC pairs exhibited more binding sites than expected through chance in all 5 regions tested. This result contradicts the idea that miRNAs can increase gene expression by binding to promoter or enhancer regions. Our data suggests that any increased expression due to the binding of miRNAs to mRNAs or flanking regulatory elements is either very rare or undetectable by our method (perhaps because they function at a translational level).

**Figure 3:**
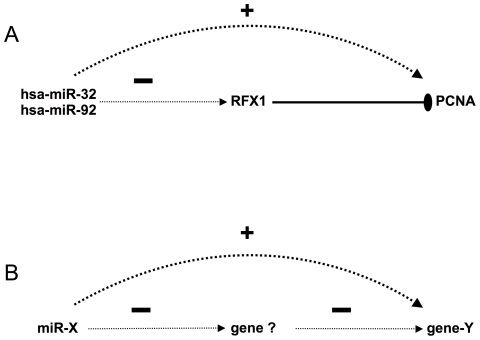
Positive correlations between miRNA and mRNA can be explained by intermediates. Solid lines represent published regulatory relationships, whereas dotted lines represent CPC pairs (+) and CNC pairs (−). A. hsa-miR-32 and hsa-miR-92 (Figure 2B) repress RFX1 via a 3′UTR sequence. RFX1 represses PCNA [29]. This results in a positive correlation in expression between hsa-miR-32, hsa-miR-92 and PCNA. B. Systematic discovery of indirect regulation where a putative “intermediate” gene (“gene ?”) explains the positive correlation between the miR-X and gene-Y. For each pair, we search for a direct target of miR-X, which is also an inhibitor of gene-Y. This was achieved by identifying genes that were negatively correlated with both miR-X and gene-Y.

Although our analysis was not designed to identify a mechanism by which miRNAs increase mRNA expression, many miRNA/mRNA pairs exhibited unexplained high levels of CPC. To further explore this substantial family of CPC pairs, we focused on the PCNA gene (proliferating cell nuclear antigen) involved in cell replication and DNA repair because it was highly positively correlated with both hsa-miR-92 and hsa-miR-32. To explain the positive correlation between PCNA and the two miRNAs, we hypothesized that one or many other genes could be inhibited by miR-92 and miR-32 and that these genes could be negative regulators of PCNA (Figure 3A). This “intermediate” regulation could explain the positive correlation between the two miRNAs and PCNA. Interestingly, a known inhibitor of PCNA transcription is Regulatory Factor X 1 (RFX1) [29]. To test if hsa-miR-92 and PCNA were positively correlated because of the effect of RFX1, we performed the luciferase assay (Figure 2B). This experiment showed for the first time that hsa-miR-92 targets the 3′UTR of the RFX1 transcript, which is in turn known to inhibit PCNA expression. This relationship explains the positive correlation found between hsa-miR-92 and the PCNA gene. To investigate how many CPC pairs could be explained by this type of indirect regulation, we searched for “intermediate” genes such as RFX1 that were negatively correlated to both the miRNA and to the mRNA in a CPC pair (Figure 3B). Amongst the 1717 CPC pairs, we found that 740 were linked via a predicted “intermediate” gene that was negatively correlated (>−0.3) with both the mRNA and the miRNA of the pair (Table S6). Amongst the 740 “intermediate” genes, 136 were identified using Gene Ontology [30] as negative regulators of transcription (GO:0016481) with a highly significant enrichment in this gene category (9.2E-10 with Benjamini correction, DAVID Functional annotation tool [31]). When the same analysis was performed on putative intermediate genes with weaker correlations (−0.1<r<−0.3) with the miRNA in a CPC pair, the enrichment was no longer significant (P = 0.07). Moreover using the same approach for discovering putative binding sites as described in the energy walk, we found that there were significantly more putative targets between the miRNAs and the intermediate genes annotated as negative transcription regulators than between the same miRNAs and the mRNA from the CPC pair (71/136 versus 32/136, P = 0.001, Fisher's exact test). Although alternative hypothesis can explain the correlation between CPC pairs, we believe that our results taken together, point towards the widespread indirect regulation of transcription by miRNAs targeting transcription inhibitors. This effect may explain the high number of CPC pairs identified in our dataset. Surprisingly, the number of CPC pairs is 23% higher than the number of CNC pairs indicating that indirect targeting of miRNAs is a major effect that should be considered with equal importance to direct targeting. Complex examples of indirect regulation through miRNAs have already been described [32], however we report for the first time an *in-silico* approach capable of detecting and quantifying indirect regulation by miRNAs.

The statistical significance of CPC and CNC pairs does not necessarily allow us to conclude that a given miRNA regulates the mRNA. Both members may be subject to regulation by external factors that lead to concerted or opposite expression patterns of the miRNA and the mRNA. We suggest that investigators search for sequence complementarity between the miRNA seed region and the 3′UTR of putative targets before validating CNC pairs in a reporter assay as we have done in this study. Interestingly our analysis was capable of detecting miRNAs regulated by mRNA genes. One example of this is the CPC pair hsa-miR-146a and RELA. RelA protein is a subunit of the NF-kappaB complex that has been identified as an enhancer of hsa-miR-146a [33]. This enhancer effect most likely explains the positive correlation between the RELA mRNA and hsa-miR-146a. In conclusion, although conserved correlation is insufficient to ascertain direct regulation of protein coding genes by miRNAs, this novel approach is capable of discovering functionally related miRNA/mRNA pairs.

Our approach is limited by the amount and quality of publicly-available expression data from different organisms and in different tissue and cell types. Many tissue specific miRNA/mRNA pairs could not be tested because expression data from their cognate tissue type was unavailable. Surprisingly, we were able to predict and confirm CNC pairs with tissue specific miRNAs such as miR-124. Although this miRNA is specific to brain, its expression was measured in many subtypes of brain tissue allowing us to calculate correlation coefficients between miR-124 and different mRNAs. We believe that this conserved correlation approach will become increasingly popular as deep sequencing technologies increase the amount of available expression data in multiple tissue types, organisms and developmental stages [34]. This approach can easily be extended to the discovery of novel interactions between mRNA genes and other functional RNA molecules, the majority of which are suspected to play key roles in many biological processes [35],[36].

## Discussion

In this study, we showed that combining expression data from human and mouse could effectively predict genes that are regulated by miRNAs through direct targeting or through an indirect effect. This approach alleviates the problems of noisy data from experiments that involve measurement of expression and thereby allowed us to infer functional relations between miRNAs and target genes. Not only were we able to detect new miRNA targets with this approach, we were also able to identify indirect targeting that leads to positive regulation of gene expression. This positive regulation does not function through the binding of a miRNA to its target but through intermediate molecules such as transcription inhibitors and may be even more prevalent than direct inhibition of messenger targets. Because our approach does not rely on the knowledge of a specific mode of action, it can be widely applied to other families of functional RNA molecules.

## Materials and Methods

### Expression data selection and processing

Expression data of human and mouse miRNAs with a total clone count > = 30 were downloaded from the miRNA expression atlas [16]. This filter allowed us to eliminate miRNAs for which the clone count never exceeded 2 in a given tissue or cell type and were therefore unlikely to play a major role in the samples examined in this study.

We analyzed 120 experiments from the hgu133a platform for human and 75 experiments from the 430_2.0 platform for mouse. We chose these platforms because they have been the most extensively used according to the Gene Expression Omnibus (GEO) [37] and cover the widest range of tissue types. Our selection of microarray data consisted in retrieving experiments performed on the same tissue types as those listed in the miR atlas according to their GEO descriptions. For each tissue type, we selected 3 microarray experiments from independent studies (this was not possible for 2 tissue types in human and 5 in mouse due to lack of sufficient experiments: Table S1 and S2). Having verified that each experiment from the same tissue type had a Pearson's correlation coefficient above 0.9 with the 2 other experiments after preprocessing steps, we selected the experiment with the highest correlation coefficient with the 2 other experiments. If an experiment was not in agreement with the 2 others (corr. coeff <0.9), the experiment was discarded and replaced by a new experiment upon which the procedure was repeated. We therefore collected microarray experiments that were highly representative of the tissue types studied.

Microarray expression data was retrieved from the celsius server [38] through R scripts (http://cran.r-project.org/). The Celsius server provides scripts for querying, and exporting primary and pre-processed Affymetrix microarray data. All array data was imported preprocessed with the RMA (Robust Multichip Average) expression measure [39].

### Orthologous mRNA gene and miRNA mapping

Orthologous probes between human and mouse were identified (16690 common probes) and mapped to their corresponding gene symbol (6920 unique gene symbols) using the Resourcerer webtool [40]. In cases where multiple probes mapped to the same gene, the same iterative procedure as described above for microarray experiments was applied to identify the most representative probe. Orthologous miRNAs were identified by name in the Atlas [18]. In total, our dataset contained expression measurements of 117 orthologous miRNAs and 6920 orthologous protein coding genes from 35 different human, and 28 different mouse samples (Tables S1, S2 & S3).

### Enrichment analysis

An enrichment analysis was performed by comparing the relative overlap between negatively correlated pairs from each bin and predicted miRNA/target-mRNA pairs from either TargetScan (4.1) or miRBase (v11) databases. The relative overlap was calculated using the hypergeometric distribution. This distribution describes the number of successes in a sequence of *n* draws from a finite population without replacement. Here, *n* draws was the number of CNC pairs in each bin and the number of successes was the number of miRNA/mRNA pairs common to the CNC bin and the database considered. The *n* draws were taken from the finite population of all possible combinations of the 117 miRNAs and 6920 genes (809,640). Using this background model instead of considering combinations of all known mRNA and miRNA genes ensured that any enrichment found in this analysis was not due to restricting our dataset to orthologous mRNA and miRNA genes. Lower p-values, and thus higher bars in Figure 1, correspond to higher levels of relative overlap between negatively correlated pairs from each bin and predicted miRNA/target-mRNA pairs from each database.

### Luciferase assays

3′UTR sequences and pri-miRNA sequences were retrieved from the Ensembl database (http://www.ensembl.org). The segments of the 3′UTRs containing the miRNA binding site were amplified by PCR from normal human genomic DNA using Phusion (Finnzymes) and cloned into the pGEM-T-Easy (Promega) intermediate vector for sequence confirmation. The 3′UTR sequences were cloned into the pSiCHECK2 vector (Promega) downstream of the renilla luciferase gene using the NotI site. The vector also carries the firefly luciferase gene for normalization. Mutant plasmids were generated by PCR from the pGEM-WT plasmid using Phusion and primers carrying seed-site mutations. All final normal endogenous and mutant plasmids were confirmed by sequencing. Pri-miRNA sequences were amplified from genomic DNA with primers carrying an XbaI site on the 5′ and an AgeI site on the 3′ primer. The products generated were the pre-miRNA hairpin with ∼100 bases flanking either side. The pri-mirs were cloned into the pLKO vector (SIGMA) and the sequences were confirmed.

Adherent HeLa cells (ATCC: CCL-2) were grown in DMEM supplemented with 10% fetal calf serum and antibiotics. Cells were plated at 6–8×10^4^/well in 24-well plates one day prior to transfection, at which point they had reached 80%–90% confluency. The cells were transfected with the pSiCHECK2 plasmid (50 ng) and the miRNA overexpression PLKO plasmid (100 ng) in a final volume of 0.5 mL using Lipofectamine 2000 (Invitrogen). Firefly and Renilla luciferase activity was measured consecutively using a Dual Luciferase Assay Kit (Promega) 24 h after transfection. Each plasmid was tested in three independent experiments, each performed in triplicate (nine transfections in total). Renilla luciferase values were normalized to the firefly luciferase values by division.

### Energy walk

We analyzed 5 regions for each mRNA in CPC and CNC pairs to perform the energy walk. Enhancer sequences were downloaded from the VISTA website [23]. 5′UTR, 3′UTR, coding and the 3 kb upstream sequences (Transcriptional Start Sites) were downloaded from Ensembl [41] (Release 47) using the Ensembl perl API scripts. When a gene contained multiple transcripts (variable 3′UTR, 5′UTR or alternative splicing isoforms), we created a chimeric transcript with the longest 3′UTR and 5′UTR sequence and an assembly of all exons in the different transcripts. This ensured that potential binding sites in alternative mRNA isoforms would be detected. Sequences from these 5 regions were scanned using a sliding window of 25 bp with a 5 bp step, considering both strands for VISTA and 3 kb upstream regions to detect DNA binding. The binding energy between the sequences in each window and the selected miRNA was calculated using a Free energy calculation with the Vienna package as described in [42]. If the binding energy in one window was <−20 KCal, then the region studied was considered to have a high energy binding site for the miRNA considered. The shuffled data was produced by reassigning the miRNAs involved in a CPC or CNC pair to another randomly selected mRNA from another pair. Fisher's Exact Test for Count Data was used to verify the significance between the number of high energy binding sites in the real data and the shuffled data. To eliminate pairs positively correlated because of a common cis-acting element, we discarded CPC pairs whose genomic coordinates were within 100 kb of each other. Amongst the 1735 CPC pairs, 18 were expressed from the same genomic locus (including the well documented hsa-miR-10a-HOXB5 and hsa-miR-196a-HOXB7 pairs [43],[44].

## Supporting Information

Figure S1 Receiver operating characteristic (ROC) curve comparing the efficiency of using negatively correlated miRNA/mRNA pairs (809,640 pairs) in human (blue) and conserved negatively correlated pairs between human and mouse (red). Pairs from both groups were ordered by their r value and split up into 100 groups of increasing size (increments of 8096 pairs). For each group we measured the number of pairs predicted to be miRNA targets by TargetScan or miRBase. The y-axis represents the number of overlapping pairs as a proportion of the total number targets predicted by one of the 2 algorithms for the 809,640 pairs. The x-axis represents the number of non-overlapping pairs as a proportion of the total number targets predicted by one of the 2 algorithms for the 809,640 pairs. A unique conserved correlated r value was calculated for conserved pairs by transforming the r values into z scores, taking the mean of these transformed scores and recalculating an average r from this z score. This ensures that sample size and distribution is accounted for (Silver et al., Journal of applied Psychology. 1987).
(0.44 MB TIF)

Text S1 Comparison of conservation levels in negatively correlated pairs and conserved negatively correlated pairs (CNC).
(0.03 MB DOC)

Table S1 Microarray data selected for human mRNA.
(0.12 MB XLS)

Table S2 Microarray data selected for mouse mRNA.
(0.12 MB XLS)

Table S3 Clone counts for mature human microRNAs.
(0.16 MB XLS)

Table S4 High scoring CNC pairs.
(0.12 MB XLS)

Table S5 miRNA transfection experiments.
(0.11 MB XLS)

Table S6 CPC pairs linked via a predicted “intermediate” gene.
(0.18 MB XLS)
