# Supplementary figures and images for: Conserved Expression Patterns Predict microRNA Targets
Source: PLoS Comput Biol. 2009 Sep 25;5(9):e1000513. doi: 10.1371/journal.pcbi.1000513 (PMC2736581; doi:10.1371/journal.pcbi.1000513)

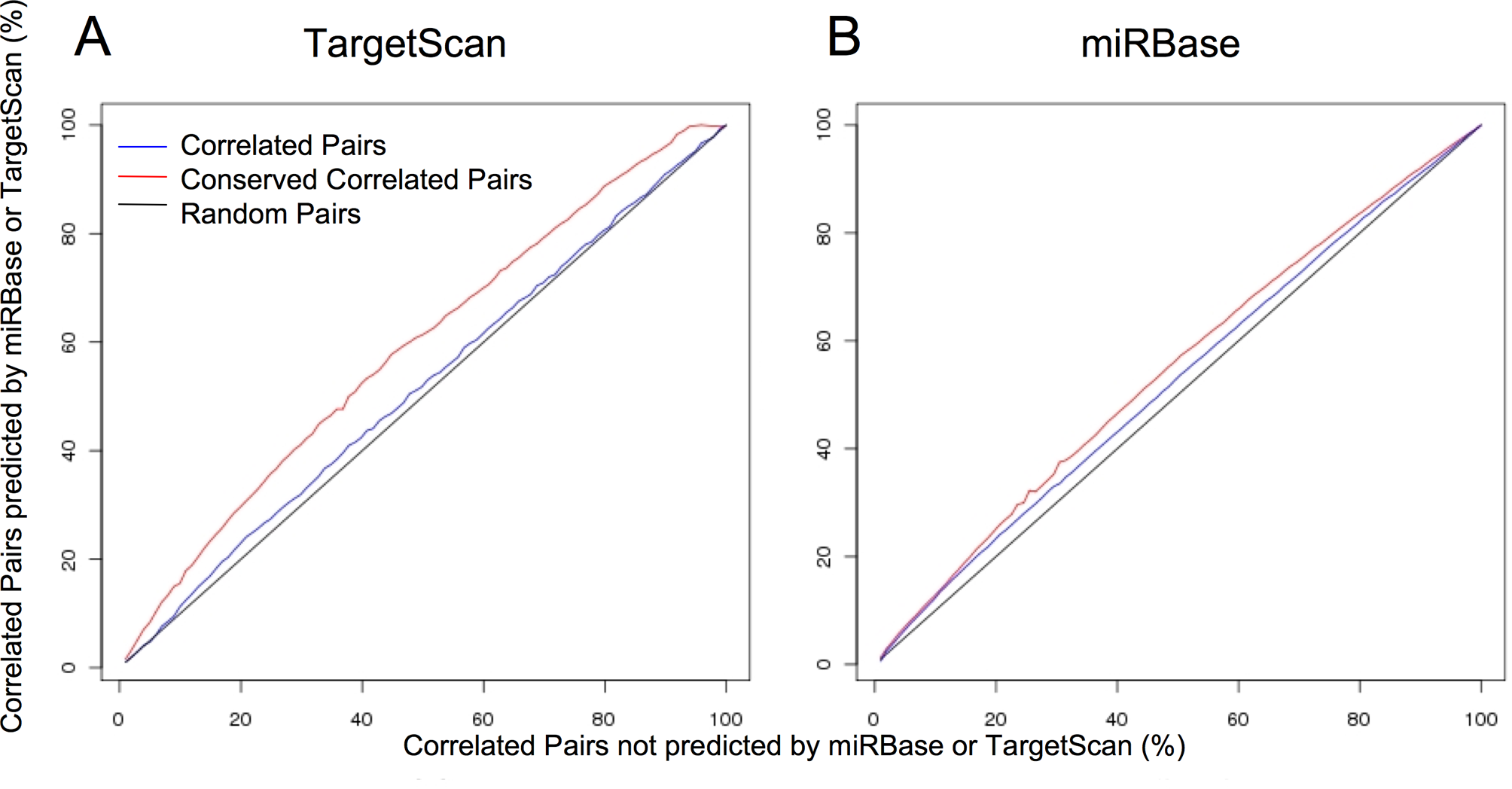

Supplement: Figure S1 — Receiver operating characteristic (ROC) curve comparing the efficiency of using negatively correlated miRNA/mRNA pairs (809,640 pairs) in human (blue) and conserved negatively correlated pairs between human and mouse (red). Pairs from both groups were ordered by their r value and split up into 100 groups of increasing size (increments of 8096 pairs). For each group we measured the number of pairs predicted to be miRNA targets by TargetScan or miRBase. The y-axis represents the number of overlapping pairs as a proportion of the total number targets predicted by one of the 2 algorithms for the 809,640 pairs. The x-axis represents the number of non-overlapping pairs as a proportion of the total number targets predicted by one of the 2 algorithms for the 809,640 pairs. A unique conserved correlated r value was calculated for conserved pairs by transforming the r values into z scores, taking the mean of these transformed scores and recalculating an average r from this z score. This ensures that sample size and distribution is accounted for (Silver et al., Journal of applied Psychology. 1987). (0.44 MB TIF) [file pcbi.1000513.s001.tif]
