# Supplementary material for: Conserved Expression Patterns Predict microRNA Targets
Source: PLoS Comput Biol. 2009 Sep 25;5(9):e1000513. doi: 10.1371/journal.pcbi.1000513 (PMC2736581; doi:10.1371/journal.pcbi.1000513)
Supplement: Text S1 — Comparison of conservation levels in negatively correlated pairs and conserved negatively correlated pairs (CNC). (0.03 MB DOC) [file pcbi.1000513.s002.doc]

**Comparison of conservation levels in negatively correlated pairs and conserved negatively correlated pairs (CNC).**

To verify that the high level of overlap between TargetScan (Figure 1), that relies on conserved seed sequences, and CNC pairs is not due to the fact that genes with similar expression patterns may have higher sequence conservation in the 3’UTR region, we performed the following experiment. We collected 3’UTR sequences of 1393 mRNA genes from CNC pairs with a *r* < -0.3 and from a control set of 1393 randomly selected genes amongst the 9570 with a *r* <-0.3 in human but *r* > -0.1 in mouse. We then verified the degree of sequence conservation between the two sets to see if the 3’UTR sequence of negatively correlated pairs in human and mouse were more conserved than those from negatively correlated pairs in human but not in mouse. For each mRNA we downloaded 3’UTR genomic sequences of each alternative transcript from Ensembl using BioPerl scripts. We then measured conservation using the genomic evolutionary rate profiling (GERP) algorithm (Cooper et al., 2005). GERP identifies conserved regions and estimates evolutionary rates for individual alignment columns. We used pre-calculated GERP scores generated by ENSEMBL on a 10-way alignment of amniota vertebrates (Flicek et al., 2007) for both sets of 3’UTR sequences. The average conservation scores for the two sets were 1.188 and 1.191 respectively with no statistical difference between them (p>0.05, two-sided, unpaired t-test). This results indicates that the higher levels of overlap between high scoring CNC pairs and TargetScan is not due to globally higher levels of conservation in the 3’UTR of orthologous genes with similar expression patterns.
